# Novel Treatment of Vomiting of Pregnancy

**Published:** 1875-08

**Authors:** Edward Copeman


Novel Treatment of Obstinate Vomiting in Pregnancy.—*By Edward Copeman, M.D., F.R.C.P.*—On June 9th, 1874, I was summoned to a lady, thirty-five years of age or thereabouts, to consult with two other practitioners already in attendance. She was about six months gone in pregnancy, and was so reduced by almost incessant vomiting that great fears were entertained as to her safety. I noticed there was slight uterine action accompanying the sickness, and, on examination, I found the os uteri partially dilated so as readily to admit the finger. I thought it right under the emergency to advise bringing on labor without delay; the gentlemen present, however, expressed no little apprehension as to whether or not she would have strength to undergo the effort of parturition on account of the very depressed and exhausted state of her system. They nevertheless concurred in the advisability of the course I recommended, and asked me to perform the operation. I at once dilated the os uteri as much as I could with the finger, and could feel the membranes and the head of the child. I tried to rupture the membranes with a telescopic female catheter (the only instrument at hand,) but they were so placid and the head offered so little resistance, the catheter shortening itself also on my making pressure, that I could not succeed; and, thinking it wise to wait awhile before resorting to any other expedient, we retired to another room for further consultation. In about one hour we saw the patient again, and were surprised to find that a longer period had elapsed without sickness than before; and we again waited, in the hope that she might be able to take a little nourishment, and so be better prepared to undergo any further proceeding. We waited another hour, but there was no return of the vomiting; and we spent the rest of the night in watching, during the whole of the time she was improving, and we determined to let well alone. I left her early in the morning, and had a favorable account of her a few days afterwards. There was no return of sickness; she went on to the full period of pregnancy, was then delivered of a healthy child, and made a good recovery.—*Clinic.*

